# Why the MDGs need good governance in pharmaceutical systems to promote global health

**DOI:** 10.1186/1471-2458-14-63

**Published:** 2014-01-21

**Authors:** Jillian Clare Kohler, Tim Ken Mackey, Natalia Ovtcharenko

**Affiliations:** 1 Leslie Dan Faculty of Pharmacy, University of Toronto, 144 College Street, Toronto M5S 3 M2, ON, Canada; 2 Department of Anesthesiology, University of California San Diego School of Medicine, 200 West Arbor Drive, San Diego, CA 92103 USA

**Keywords:** Good governance, Corruption, Health systems, Pharmaceutical systems, Access to medicines, Global institutions

## Abstract

**Background:**

Corruption in the health sector can hurt health outcomes. Improving good governance can in turn help prevent health-related corruption. We understand good governance as having the following characteristics: it is consensus-oriented, accountable, transparent, responsive, equitable and inclusive, effective and efficient, follows the rule of law, is participatory and should in theory be less vulnerable to corruption. By focusing on the pharmaceutical system, we explore some of the key lessons learned from existing initiatives in good governance. As the development community begins to identify post-2015 Millennium Development Goals targets, it is essential to evaluate programs in good governance in order to build on these results and establish sustainable strategies. This discussion on the pharmaceutical system illuminates why.

**Discussion:**

Considering pharmaceutical governance initiatives such as those launched by the World Bank, World Health Organization, and the Global Fund, we argue that country ownership of good governance initiatives is essential but also any initiative must include the participation of impartial stakeholders. Understanding the political context of any initiative is also vital so that potential obstacles are identified and the design of any initiative is flexible enough to make adjustments in programming as needed. Finally, the inherent challenge which all initiatives face is adequately measuring outcomes from any effort. However in fairness, determining the precise relationship between good governance and health outcomes is rarely straightforward.

**Summary:**

Challenges identified in pharmaceutical governance initiatives manifest in different forms depending on the nature and structure of the initiative, but their regular occurrence and impact on population-based health demonstrates growing importance of addressing pharmaceutical governance as a key component of the post-2015 Millennium Development Goals. Specifically, these challenges need to be acknowledged and responded to with global cooperation and innovation to establish localized and evidence-based metrics for good governance to promote global pharmaceutical safety.

## Background

The potential for corruption to limit human development has gained traction within the global development community since then World Bank President James Wolfensohn’s 1996 speech on the “cancer of corruption” [1]. As the conversation on corruption has progressed, attention has shifted to how it can be prevented, with an emphasis on good governance. Since then, there have been a number of important milestones, including most notably, promotion of good governance as a core element in the post-2015 development agenda carrying forward the United Nations Millennium Development Goals (MDGs) [2].

Progress has also included the development of widely accepted international binding norms recognizing the importance of coordinating global action against forms of corruption. In October 2003, the United Nations General Assembly adopted the United Nations Convention against Corruption (UNCAC) under the United Nations Office of Drugs and Crime. The UNCAC is an international treaty instrument that specifically addresses the prevention, criminalization, and mobilization of international cooperation in support of anti-corruption activities. The engagement of diverse international organizations and civil society actors has been crucial in highlighting the overall impact of corruption on development and identifying broad strategies to address it [3].

However, to be successful, generally good governance programs addressing corruption must be more focused in their target areas. This often includes assessment of diverse elements of both the public sector and the private sector environment. In addition, sectors such as health systems are highly complex and require tailored governance approaches to identify potential risk factors associated with corruption, waste, and fraud and abuse. For example, the delivery of life-saving pharmaceutical commodities requires specific training and knowledge unique to this critical component of the global, national and local health infrastructure. In order to inform future efforts and establish better causality between good governance initiatives and health outcomes in this sector, careful examination and comparison of existing initiatives attempting to address this issue is essential. This will ensure that future work in pharmaceutical good governance is appropriately informed by evidence and lessons learned, increasing the likelihood of continued success and improvement.

Certain aspects of good governance may be mostly relevant to lower and middle-income countries where governance is deficient in national health systems. This includes governance areas of maintaining regulatory impartiality, ensuring quality and safety of medicines in manufacturing and procurement practices, and preventing undue pay-offs for health care services. Others are highly relevant in high-income countries such as preventing fraud and abuse and preventing illegal marketing practices of pharmaceutical companies.

For example, the United States, the largest pharmaceutical market in the world, has experienced record breaking criminal and civil fines associated with illegal pharmaceutical marketing, including some USD$4 billion in recoveries in 2010 [4]. Illegal pharmaceutical marketing in the United States has been uncovered by whistle-blowers, likely helped by enabling U.S. legislation that includes certain protections and incentives, and have revealed practices that led to increased drug costs, biasing or misrepresenting scientific evidence/education, and direct risks to patient safety through illegal off-label promotion [4,5]. This includes a recent settlement against Johnson & Johnson of USD$2.2 billion for misleading doctors about the safety of its antipsychotic Risperdal [6,7]^a^. It also illuminates the importance of structural changes that, if implemented and monitored, ensure better governance. As an example, even though we are finding an increase of physician-directed pharmaceutical marketing transparency laws internationally, continued violations and legal settlements indicate that more rigorous governance initiatives may be needed [8].

As an initial commentary on existing governance initiatives in the pharmaceutical sector where the public and private sphere often intersect, this paper identifies three thematic areas whose effective management is essential for success: authentic country ownership of good governance initiatives, understanding about how the political context may help or hinder an initiative, and monitoring and evaluating how good governance initiatives may help improve health outcomes. We conclude with a discussion on how international cooperation and shared governance approaches that emphasize improvement in these areas can promote global pharmaceutical safety and advocate for their need in the future development of the MDGs.

### Importance of good governance in health

As a broad concept, good governance implies a system which is consensus oriented, accountable, transparent, responsive, equitable and inclusive, effective and efficient, follows the rule of law, and is participatory [6,7]. Corruption, which is defined by the World Bank as the “abuse of public office for private gain”, is assumed to be more likely when good governance is not present although establishing causality is not straightforward in practice [8]. We use the World Bank’s definition for our paper as we specifically examine why pharmaceutical governance is important for equitable and efficient access to medicines. While the focus of our paper is good governance in the public sector and in international agencies and development initiatives, we do acknowledge the obvious importance of examining this issue in the private sector as well. This includes additional exploration of industry-specific prevention measures in health governance mechanisms to improve transparency, anti-corruption and anti-bribery tools, industry regulation, and legal prosecution of illegal practices.

In order to better explore the relationship between health governance and corruption, there is a clear need for better data collection and agreed upon metrics to support research and evidence-based policymaking. In response, several organizations including the World Bank, Transparency International, and the World Justice Project have attempted to develop indicators and indexes to better assist states in improving governance, controlling corruption, assessing the rule of law, and minimizing potential negative impact from these factors in development projects. However, divergent institutional approaches, varying indicators across organizations, and a lack of robust research addressing the underlining relationship between governance and corruption, currently pose challenges to promoting anti-corruption activities, especially in the health sector.

In the past decade, available research has provided increasing evidence that greater vulnerability to corruption can lead to limiting access to medicines and health services [3,9]. In Venezuela, for example, approximately two-thirds of hospital personnel surveyed were aware of theft of medical supplies and medications [10]. Similarly, in Costa Rica, 71 per cent of doctors and 83 per cent of nurses reported that equipment or materials had been stolen in their hospital [11]. One study in Uganda found that the resale of drugs represented the greatest single source of income for health care personnel [12]. Another study by Amnesty International on maternal health in Burkina Faso found that one of the primary causes of annual mortality in thousands of pregnant women (including during childbirth) is due to corruption by health professionals and poor healthcare delivery [13]. Poorer women may also lack access to critical health care services simply because they are unable to pay informal fees [13]. Collectively, these case studies identify key forms of corruption in the pharmaceutical and healthcare delivery infrastructure, often exacerbated by a lack of financial resources, poor remuneration of healthcare professionals, and the brain drain of healthcare workers [14]. However, identification and evaluation of health corruption may fail to explicitly measure key governance indicators leading to a lack of evidence regarding the direct relationship between lack of good governance and its impact on enabling corrupt practices.

Conversely, evidence has also shown that good governance can improve key development goals; a study from Transparency International demonstrated that increasing transparency, accountability and integrity in 48 countries had a robust correlation to better outcomes in health, education and water access [15]. Another example is program evaluation efforts by the Inter-American Development Bank (IDB) that conducted an external audit of public hospitals in Bogota, Colombia analyzing the current situation of hospital finances, highlighting problematic areas, creating action plans to resolve issues uncovered, and implementing a monitoring and evaluation process with dissemination of information related to this project through workshops. This external review provided Bogota’s Secretariat of Health with evidence that fraud was taking place, and in response measures were taken to reduce theft and improper billing in the hospital setting [15]. Another clear example of how good governance generates benefits is with pricing transparency. The public posting of medical supplies purchased by public hospitals by the city government of Buenos Aires, Argentina resulted in prices reductions within the first few months of this intervention. Arguably, the mere anticipation of price reporting/transparency helped lower prices. Unfortunately the price declines were not maintained over time, suggesting the importance of designing multi-prong anti-corruption strategies that reinforce these selective responses over time [16].

Despite this growing recognition of the importance of good governance in health, corruption continues to be reported in national and global health systems across a range of environments and has undermined progress in health services delivery and system strengthening at a time when investments in global health interventions continue to be strong but also heavily scrutinized [4,17]. This includes a diversity of forms of health-related corruption in both the private and public sphere in high-income countries, developed country settings, and in multilateral initiatives. These forms of corruption have yet to be effectively addressed through a global governance framework specific to health-related corruption or pharmaceutical governance [4].

### The need for good governance in the pharmaceutical system

The efficient and safe selection, procurement, storage and delivery of pharmaceutical commodities are essential functions in ensuring sustained global health outcomes, especially in resource-poor settings. Good governance approaches and absence of corruption in both the public and private pharmaceutical sector are crucial in maintaining adequate distribution of essential medicines for populations who lack access, preventing diversion/theft of pharmaceuticals, protecting against illegal pharmaceutical promotion (e.g. illegal off-label promotion), and ensuring that medicines are safe and not falsified [18,19]. For instance, an example of industry conflicts of interest is apparent in a recent decision made by a USA Food and Drug Administration (FDA) Advisory Committee (with members that included those with ties to the manufacturer of the oral contraception products under review) to keep birth control pills Yaz and Yasmin on the market, even though they were associated with increasing patient adverse safety events.

In addition, with increasing prevalence and attention to non-communicable diseases globally (highlighted by the recent 2011 United Nations High-level Meeting on Non-communicable Diseases) that often require sustained pharmaceutical treatment, coupled with annual global pharmaceutical spending that is forecasted to reach USD$1.2 trillion by 2016, the integrity of the global pharmaceutical supply chain is clearly important to the future of global health [20]. Yet, despite this increasing need and rapid market growth, pharmaceutical good governance in individual countries has been markedly uneven globally.

When good governance is absent in the pharmaceutical system, access to and quality of medicines are affected [21]. Hence, good governance in pharmaceuticals should ideally be a guiding principle to the operation of the entire pharmaceutical supply chain and health delivery system. This is particularly important in the context of ensuring progress is made towards reaching the MDGs and as post-2015 targets are established. In fact, recognition that good governance must be an essential component of all programs focused on the health system is necessary within the context of promoting access to medicines as a human rights issue, a concept that has been reinforced by resolutions and reports issued by the UN General Assembly, UN Human Rights Council, and World Health Organization (WHO).

Importantly, there is no single “prescription” to entrench good governance in the pharmaceutical sector. The sector presents unique challenges: demand is often greater than the supply, there is significant uncertainty in how much of each product is needed, and the complex globalized drug supply chain comprised of both public and private sector actors has the potential for market failure at each step. It is clear, however, that these challenges cannot become excuses for inaction. Rather, multiple approaches and possible global efforts aimed at adapting to local requirements, while at the same time attempting to achieve some measure of harmonization in procurement and delivery for purposes of efficiency, must continue to be explored. These steps are necessary in order to enact effective change at the national and global level informed by examination of strengths and weaknesses of current pharmaceutical governance initiatives.

E-procurement as done in the Chilean procurement system is often cited as an effective tool to prevent corruption in drug procurement. Through the use of electronic bidding and information dissemination about procurement procedures and results, corruption has been curbed substantially. These anti-corruption interventions were even more effective when a wide range of suppliers competed for each product openly so that price competition was fostered and opportunities for collusion were reduced [22]. Such examples of innovative forms of governance and technology utilization should be encouraged and incorporated into future pharmaceutical governance initiatives and may have shared value among stakeholders.

### Current pharmaceutical governance efforts

Recognizing that good governance matters for improved health outcomes and return on development investments, global institutions like the World Bank, the WHO’s Good Governance for Medicines Programme (GGM), the Global Fund for HIV/AIDS, Tuberculosis, and Malaria (Global Fund), and the UK Department for International Development (DFID) through the Medicines Transparency Alliance (MeTA) have launched a number of initiatives in the past decade^b^. These programs have used a range of approaches, with varying levels of success but are primarily focused on low to middle income country settings and public sector governance improvements. Each initiative has now been in operation for enough time that it is possible to provide a preliminary review of their impacts and draw lessons for future development^c^.

### The World Bank

The World Bank has addressed good governance in a number of ways and was an early pioneer of measuring good governance in the pharmaceutical system by developing a tool to determine vulnerability to corruption in 2002. Currently, procurement practices are monitored on a grant-by-grant basis whenever medicines purchases are required [23]. Still, in 2007-2008, fraud and corruption were identified in procurement for a major project in India [23]. This led to unintended negative consequences. For example, policy discussions were significantly hampered as well as investments in health systems projects. However, more positively, these findings of fraud and corruption solidified efforts by the World Bank to improve its country-client procurement practices [21]. The World Bank, however, is less focused on anti-corruption interventions in the pharmaceutical system *per se* but rather on broader health systems strengthening projects, which may have a less discernible result in terms of their specific impact on good governance in the pharmaceutical system.

### WHO’s Good Governance for Medicines Programme (GGM)

GGM, at the time of this writing has been in place in 36 countries; it emphasizes strengthening rules and procedures within the supply chain and on instilling a long term government commitment to good governance [24]. The program begins by assessing a country’s pharmaceutical system’s vulnerability to corruption, using a tool based on one that was developed by the World Bank. Based on the results, a plan to strengthen good governance is established and then implemented in partnership with the national government. This model uses a fairly rigid structure in the early stages of the project, measuring all countries on the same criteria, but flexibility increases as countries move through to the policy change stages. Following a reassessment of countries (using the same vulnerability to corruption methodology), it was found that the improvements in good governance were made predominately in registration, drug selection and procurement and were less apparent in areas such as clinical trials and marketing practices.

### Medicines Transparency Alliance (MeTA)

In its pilot phase (2008-2010), MeTA was put in place in seven countries (the Philippines, Zambia, Jordan, Kyrgyzstan, Peru, Uganda and Ghana) [25]. Phase II (2012-2016) is in place in the same countries, although entry and exit criteria have been established to accommodate any future changes. MeTA emphasizes the disclosure of information as a way to improve transparency, accountability, and ultimately access to medicines. The premise of MeTA is that transparency is essential for addressing corruption and that the movement towards greater transparency would be more effective with communication between representatives from the public sector, private sector and civil society. This resulted in the requirement for a number of disclosure surveys early in the MeTA process and the establishment of a multi-stakeholder group to lead the program in each country. Beyond these core principles, MeTA’s structure is very flexible, the establishment and implementation of work plans is flexible and elected changes are often unique to each country recognizing the need for unique local requirements. Results from MeTA included the completion of baseline surveys and data disclosure, as well as drug pricing policy changes.

### The Global Fund

The Global Fund is a grant-making organization for projects targeting the Big Three diseases (HIV, TB, and Malaria), supporting projects that will, in the long term, prevent infections and improve access to treatment [26]. Given that procurement accounts for 40 per cent of spending in Global Fund grants, ensuring that the products are of good quality and were purchased for the best possible price is essential for the Global Fund’s programming [21]. This has led to the establishment of Voluntary Pooled Procurement (VPP), allowing countries with weaker systems to purchase quality assured medicines through the Global Fund. Additionally, all purchases are submitted to a database with documents demonstrating that medicines are of good quality. While these measures have not completely prevented corruption in the granting process, it must be recognized that funds that have been identified as embezzled in subsequent investigations represent a very small proportion of the total grant portfolio. In 2010 the Global Fund’s own Inspector General identified embezzlement in a number of countries, but the total amounted to USD$34 million out of a total budget of USD$3 billion. With recent changes in leadership there have been assessments of the Global Fund’s procedures, and a comprehensive restructuring of the granting process as well as a restructuring of its procurement processes [27].

## Discussion

### Assessment of pharmaceutical governance initiatives

In addition to this discussion, a recent U4 Issue Paper [21] identifies and assesses key global good governance initiatives in the pharmaceutical sector. It outlines a number of “good enough” lessons, which can guide future program development. We highlight a few of the key ones here:

1. Donors need to promote political dialogue and stakeholder consensus building throughout the implementation of initiatives to agree on priority areas within the pharmaceutical system as well as on which tools are most effective to employ.

2. Granting institutions like the Global Fund and the World Bank should leverage their resources by providing funding to countries when good governance is in place or when there is demonstrated commitment to address governance weaknesses and to withhold resources when these conditions are not in place. Given that measuring good governance is not yet a standard government practice, this would likely require the development of pre-approval analysis of pharmaceutical systems, which should include both empirical measurements and a broader consideration of the local regulatory and political dynamics.

3. Monitoring and evaluation mechanisms need to be an essential component of any governance initiative. [There is a] need to move away from a reliance on irregular surveys and move towards better use of routine information and more “real” time sampling methodologies that can generate data to inform management decisions and sustain advocacy and vigilance by community groups.

4. Country ownership of pharmaceutical good governance initiatives is critical. This includes political support, facilitation of a dialogue with a range of local stakeholders and ensuring they are a part of program development and implementation. [This] has been an integral to the success of countries such as Jordan and the Philippines, in both GGM and MeTA. To be sure, it is complex and may be more feasible in countries that have the political and economic power to withstand any undue influence.

5. Grant-making institutions should pay more attention to making sustainable improvements in country pharmaceutical supply systems and the regulatory environment that governs them instead of [solely] on the execution of individual grants (p. 21).

However, given the diversity of approaches to good governance in the pharmaceutical system, systematic comparisons across initiatives are inherently limited. Activities are based in different institutions with distinct visions of how good governance ought to be implemented, engaged different sets of stakeholders who were involved at various degrees of commitment in different initiatives, and measurements were made using different indicators. Still, there are some core lessons that can ideally guide more effective governance work in the pharmaceutical system for the future.

To start, there is consistent evidence from all initiatives that country ownership, meaning in this context that the government where the initiative is taking place be genuinely committed to understanding what policy, institutional, and cultural reforms are needed to make good governance happen. It is also clear that multi-stakeholder engagement is critical to success. For example, the active participation of relevant stakeholders in both GGM and MeTA allowed for strong results in terms of establishing a dialogue and developing relations and trust between previously disparate groups (public sector, private sector, and civil society). In both of these programs, the inclusive development of national plans to improve governance was key, allowing those with the closest ties to specific issues to help shape and have ownership of the changes made. Granting institutions, like the World Bank and Global Fund, have strengths in these circumstances as funding can be tied to the completion of necessary changes and because of their broad membership and partnership with non-state actors. For example, the Global Fund’s new strategy specifically states in relation to one of its stated goals that, “alignment with national strategies and systems is a key principle of aid effectiveness, which contributes to enhanced country ownership, lower transaction costs, greater financial transparency, increased mobilization of multiple partners (including civil society) and sustainability – and ultimately better impact [28]”.

Conversely, changes in government, a lack of cooperation across relevant ministries, and unbalanced representation of stakeholders (for example low participation from civil society groups) are real challenges. This dependency on commitment from political leadership makes establishing sustainable programs and policies more difficult and any future initiatives must recognize that simply changing policies on paper is likely to be insufficient. It further indicates that governance initiatives must be supported with adequate transparency and rules in relation to participation of stakeholders, the establishment of formal and truly accountable cooperation mechanisms among parties, and work plans to address possible programmatic or government changes. Cultural shifts in how good governance is perceived may also be necessary for changes to be effective even if accompanied with needed changes in political commitment/leadership.

The pharmaceutical system operates as a part of an overarching and much more complex health system and this is clearly recognized by the initiatives. As noted in the case of the World Bank, pharmaceutical good governance is commonly viewed as a component of health systems strengthening programs. Cross-cutting consensus on the direction of the program and making it possible to adjust for individual country contexts is critical. It is unfortunately too common for global institutions to evade the examination of uncomfortable but critical political issues in the health sector, such as state, regulatory and policy capture as well as the global policy context. The resulting reality is that systemic change takes time, capacity, and broad commitment to implement initiatives and to determine results from them. More focused efforts on specific issues (e.g. drug registration, national formulary inclusion, or public sector generic substitution policies) can result in more rapid changes but may not result in the deeper systemic shifts necessary to improve overall system performance. The MeTA and GGM both allow for significant flexibility in how policy changes are determined, although there is more structure in GGM. Global Fund grants are driven by country ideas and the World Bank also shapes its programs based on the issues identified in countries.

Another crucial area of needed improvement in pharmaceutical governance initiatives is monitoring and evaluation. It appears that monitoring of the results from these initiatives has been relatively weak and may be influenced by the difficulty in adequately defining causal relationships between good governance reforms and health outcomes. There is an implicit assumption in the programs that by strengthening the pharmaceutical supply chain and thereby improving its governance, decreased corruption and improved access to medicines will result. The difficulty comes in identifying whether this has occurred and if access has improved, and whether it can be attributed to the improvements in good governance instead of other external factors.

In addition, procurement and quality assurance are two components of the supply chain where causality between reforms and health outcomes may be easier to establish. Quantitative-driven results can be measured in terms of reported pricing, the number of inspections and the prevalence of falsified medicines (if such data is available), and information about stock outs/availability at point of delivery. It is also feasible to determine the impact of changes such as conflict of interest policies (i.e. in the selection of essential medicines or drug formulary decisions) and deign studies to evaluate their effectiveness on improving access and affordability. Yet, monitoring and evaluation is essential if these reforms are to have legitimacy as being necessary for the strengthening of the pharmaceutical system and improving access to medicines. As evaluation procedures are developed, they must consider how to structure indicators to be informative about their actual impact on the operation of the pharmaceutical supply chain, health services, and at least to some extent the final health outcomes. These measurements will unavoidably have limitations, but while there is currently a dearth of information on how to proceed with these steps, this work is necessary if good governance reforms are to be established and sustainable and if they are to be employed more widely for the global pharmaceutical system.

## Summary

The experiences with existing initiatives reveal that efforts must be iterative, making changes when needed and also shifting focus over time for local and international requirements of the globalized drug supply chain. This is needed also in part because any change will result in different incentives and new opportunities for corruption that must be reassessed for vulnerability. This flexibility will be an essential component to future programs and long term goals for the establishment of sustainable global institutions and ensuring safe and equitable access to medicines.

Greater communication and cooperation between relevant stakeholders engaged in pharmaceutical systems, including development-granting institutions, international organizations with good governance programs, the private sector, and donor-aid recipients could support more integrated programs. This includes attempting to establish global norms on good pharmaceutical governance with the overall goal of ensuring safe and affordable access to medicines. It can also include potential harmonization of governance indicators sensitive to local needs and monitoring and evaluation instruments already developed by the World Bank, WHO, DFID and the Global Fund informed by tools and research developed by UNDP, U4 and other organizations specifically promoting good governance in pharmaceuticals.

Procurement systems can also be strengthened by leveraging efficiencies of the Global Fund VPP model and others such as UNITAID, an international organization focused on identifying innovative solutions for market failures and improving access in low-income countries. Encouraging development of innovative pooled procurement and funding for pharmaceutical commodities has the potential to help regulate and control industry driven conflicts of interest and corruption, prevent imbalances in access, trade and price negotiations, and better ensure the quality and efficacy of medicines.

Finally, increased investment and commitment to good governance in pharmaceutical programs should be a crucial component of the post-2015 development agenda and health system strengthening grants in order to strengthen overall governance, improve development efforts, end corruption, and ensure human health well-being. Without these essential components and a clear international commitment to governance in pharmaceuticals, ensuring positive outcomes in the future of global health and development cannot be assured.

## Endnotes

^a^There are various other examples of pharmaceutical companies practicing inappropriate marketing practices in the United States.

^b^These institutions and initiatives were examined because they have were focused specifically on pharmaceutical good governance and also have been implemented over a long enough period of time to generate lessons learned.

^c^These initiatives have also been the subject of a study for U4 that we noted has informed this paper.

## Abbreviations

DFID: UK Department for International Development; GGM: Good Governance for Medicines Programme; IDB: Inter American Development Bank; MDGs: Millennium Development Goals; UNCAC: United Nations Convention Against Corruption; UNDP: United Nations Development Programme; VPP: Voluntary Pooled Procurement; WHO: World Health Organization’s.

## Competing interests

The authors report no competing interests associated with this manuscript. JK was hired as a consultant to U4 and wrote a policy paper on good governance in the pharmaceutical system. This paper reflects some of the thinking from that paper’s production.

## Authors’ contributions

JCK, TKM and NO determined the main design of the article, interpretation of data, and writing of the final manuscript. All three authors read and approved the final manuscript.

## Authors’ information

JCK is an international expert in the field of global pharmaceutical policies and has worked for a variety of organizations including UNICEF, UNDP, the World Band and the WHO. She also continues to advise global institutions on global pharmaceutical policy issues. NO is a current medical student and has worked on pharmaceutical policy and access to medicines. TKM is an international expert in the field of global drug supply safety and policy addressing the falsified medicines trade.

## Pre-publication history

The pre-publication history for this paper can be accessed here:

http://www.biomedcentral.com/1471-2458/14/63/prepub
